# Strategies for Expanding Donors Pool in Heart Transplantation

**DOI:** 10.31083/j.rcm2308285

**Published:** 2022-08-15

**Authors:** Samuel Jacob, Pankaj Garg, Ishaq Wadiwala, John H. Yazji, Mohammad. Alomari, Emad Alamouti-fard, Md Walid Akram Hussain, Si M. Pham

**Affiliations:** ^1^Department of Cardiothoracic Surgery, Heart and Lung Transplant Program, Mayo Clinic, Jacksonville, FL 32224, USA; ^2^Research Department of Cardiothoracic Surgery, Mayo Clinic, Jacksonville, FL 32224, USA

**Keywords:** heart donor, donation after circulatory death, normothermic regional perfusion, bioengineered hearts, xenotransplantation, reparable hearts, hepatitis C donor, overdose donors, COVD donors

## Abstract

Heart transplant remains the criterion standard treatment for patients in end-stage heart failure. Improvement in the post-heart transplant outcomes in the last decade has contributed to increased demand for organs. Worldwide each year, more than 5000 heart transplants are performed and 50,000 people become candidates for heart transplant. In the last 50 years, there have been several attempts to expand donor criteria to increase the donor pool. Despite making hepatitis C virus, opioid overdose death, old age allowable and changing the allocation system, the gap between supply and demand is widening and unfortunately, thousands die every year waiting due to the critical shortage of organs. New technologies for heart donation after circulatory death have emerged, particularly normothermic regional organ perfusion and *ex-vivo* heart perfusion using organ care systems. However, these technologies still do not fill the gap. Continuous advancements in areas such as regenerative medicine and xenotransplantation, among others, are needed to overcome the shortage of heart donors for heart transplantation.

## 1. Introduction

There has been tremendous improvement in the management of patients with end-stage heart failure, including medical therapy and assist devices. However, heart transplant remains the criterion standard treatment for these patients [1]. Improvement in the post–heart transplant outcomes in the last 50 years has contributed to increasing demand for organs. In addition, heart transplantation is a valuable option in patients with cardiac storage diseases such as Fabry disease and cardiac amyloidosis, that increases the need of expanding the donor pool [2].

**Fig. 1.**
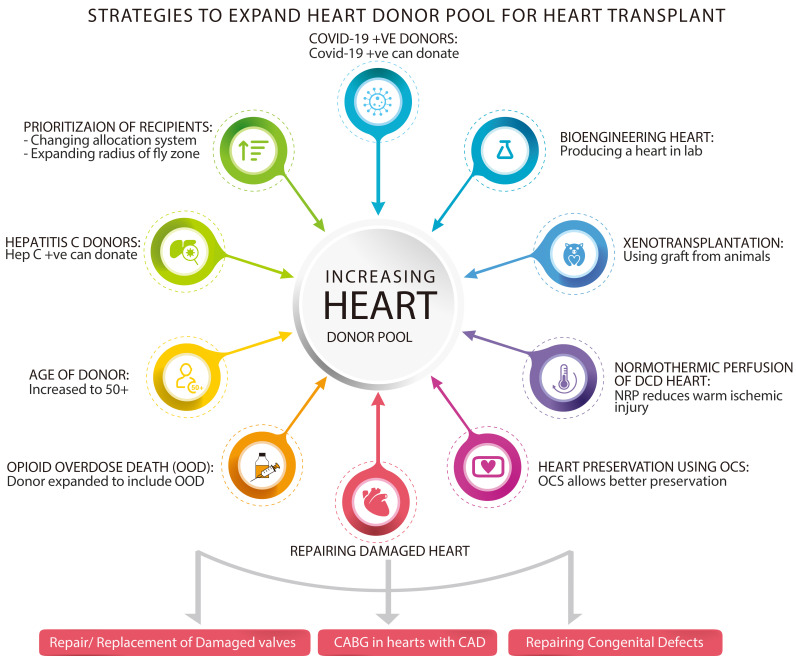
**Summarizes most of the strategies available now to expand the donors pool for heart transplantation**.

Worldwide each year, more than 5000 heart transplants are performed and 50,000 people become candidates for heart transplant [3]. In the US, more than 40,000 solid organ transplants are done annually, including more than 3000 hearts. To meet this demand, there is an urgent need to increase the potential donor pool. Presently, less than 50% of potential organ donors become actual donors [4]. In the last decade, there have been several attempts to expand donor criteria to increase the donor pool. But despite expanding donor criteria and new technologies for donation after circulatory death (DCD) heart and donor heart repair, the gap between supply and demand is widening and unfortunately, thousands die every year waiting due to the critical shortage of organs. Furthermore, even with continuous advancements in regenerative medicine and xenotransplantation [5], among other [6], to combat the need for heart transplant, the shortage of heart donors will continue to grow. The purpose of this review is to address various methods that may broaden the donor organ pool (Fig. 1).

## 2. Modifying Criteria to Expand Donor Pool for Heart Transplant

Evolution in the field of medicine has made it possible to control or treat many diseases which were once thought to be untreatable. Many potential donors who historically would have been removed from eligibility may now be considered acceptable donors. Therefore, the criteria for receiving and donating hearts needs to be updated. Expanding donor criteria will help more patients get the treatment they need and alleviate some of the stress on the waitlist pool.

The primary survey of the donor includes the confirmation of brain death, verification of consent for donation, ABO blood typing, demographics, identification of potential co-morbid conditions (including high risk behavior, substance abuse history, mechanism of death) and the need for cardiopulmonary resuscitation (and if so duration from initiation to return of vital signs) [7].

### 2.1 Hepatitis C Virus 

A sizeable number of potential donors have hepatitis C virus (HCV) due to a history of intravenous drug use. HCV has been a contraindication for transplant as the recipient would also become HCV-positive and due to poor outcomes associated with development of liver cirrhosis and hepatocellular carcinoma [8, 9]. The discovery of direct-acting anti-viral drugs, such as sofosbuvir, that have a high cure rate for HCV have sparked interest in using HCV-positive hearts for transplantation [10, 11]. A recent study by Dharmavaram *et al*. [12] showed a significant increase in the survival of patients receiving HCV-positive hearts in the past 20 years. In their study, 30-day and 1-year survival rates were similar for patients receiving HCV-positive versus HCV-negative hearts. Though long-term results are still needed, using HCV-positive hearts will substantially increase the number of available donor hearts.

### 2.2 Age

Another way to broaden the donor pool is by being more accepting of hearts from older donors. Even though age is not an absolute contraindication for heart donation, it is generally less acceptable to receive a heart from a donor older than 50 years of age. In donors aged 50 to 60 years, there is increased risk of ventricular hypertrophy, valvular lesions, and coronary artery disease (CAD). However, with careful selection of donors older than 50 years, recipient survival can be comparable to those receiving younger donor hearts [13]. Hearts from older donors with negative serologies, specifically CMV as it has been postulated to accelerate the allograft vasculopathy, normal echocardiogram and electrocardiogram, low inotropic support, normal coronary angiogram, and short ischemic time are desirable. In addition, recipient’s blood type, sex, weight, transpulmonary gradient, and pulmonary vascular resistance, should be taken into consideration. Unfortunately, there is still concern about transplanting older donor hearts into recipients older than 60 years. A study by Daniel *et al*. [14] showed poor 5-year survival in this recipient population.

### 2.3 Prioritization

Recently, a new heart allocation policy was enacted with the purpose of giving new hearts to patients who are the sickest with the hopes of it decreasing their time on the waitlist [15]. The previous allocation policy, a 3-tier system labeled 1A, 1B, and 2, had the major disadvantage of being ambiguous about which patients needed donor hearts the most [16]. The new allocation policy is a 6-tiered system that ranges from 1 to 6, with 1 being the highest priority and 6 being the lowest. With the old allocation policy, a patient with venoarterial extracorporeal membrane oxygenation and a stable patient on left ventricular assist device support would be in the same tier, while with the new policy, these patients would be in status 1 and 4, respectively [17]. A recent study by Liu *et al*. [18] demonstrated a significant decrease in the use of left ventricular assist devices and a greater likelihood of patients being supported by intra-aortic balloon pump, which resulted in fewer days on the waitlist but an increase in inpatient hospital length of stay before the transplant occurred.

Despite that new system mainly designed for optimization and prioritization the recipients who needs urgent transplantation, it has some role to expanding the pool of donors for specific transplantation groups, such as patients with rare restrictive cardiomyopathies and congenital heart disease, and the old system was inadequate sharing across geographic areas.

The new allocation system mandates organ sharing over a larger area and without regard to governmental boundaries. Status 1 and 2 candidates are now allowed to receive organs within a 500-mile radius irrespective of their donation service areas (DSA) within a UNOS Region formed the starting point from where a donor heart became available for transplant. Although this implies longer average graft ischemic time for the sickest candidates, the committee felt that the number of patients affected and the impact on post-transplant survival was likely to be small.

In addition, the new system monitor the rate of donor hearts turning down and encourage optimization and use more available hearts.

Patel *et al*. [19], recently, analyzed UNOS data which include 21,565 patients listed for transplantation, and found 14,000 met the criteria to compare the old allocation system with the new one (7035 vs 6965). The found that the new allocation system were associated with changes in O blood group in comparison to in non-O blood group, such as higher transplantation (43.8 vs 51.7) comparison to (63.4 vs 71.6), lower waitlist days (160 vs 33) compression to (77 vs 23) days, and lower waitlist mortality (5.1 vs 3.4) comparison to (4.2 vs 2.5) respectively.

### 2.4 Opioid Overdose Death

Due to the worsening opioid epidemic, there has been a rise in opioid overdose deaths (ODDs). Sadly, most patients who die from opioid overdose are younger than 45 years and greatly contribute to the donor pool [20]. According to the Centers for Disease Control and Prevention, there were over 90,000 deaths in the US related to opioid overdose in 2020 [21]. There is concern for using hearts from ODD donors due to the potential cardiac adverse effects associated with opioid use, such as hypertension, infective endocarditis, CAD, and coronary artery dissection. In a study by Dawson *et al*. [22], approximately 25% of heart failure–related hospital admissions in the US were related to prescription opioid use. Further, potential donors with history of drug use (opioid or non-opioid) may have an array of issues, including cardiovascular, respiratory, and neurologic [23]. In a study by Randall *et al*. [24], patients who received other organs, such as livers and kidneys, from donors who used opioids had significantly higher mortality and graft failure. However, in carefully selected donors with history of opioid or non-opioid drug use, long-term recipient survival can be comparable to that in recipients with organs from donors without history of drug use [25]. A more general acceptance of ODDs and other drug overdose–related deaths for heart donation will further expand the donor pool.

Latest recommendations and major criteria based on a review of the literature and international society of heart and lung transplantation (ISHLT), conducted by Sathianathan and Bhat concluded the most important criteria which include: AVOID female donors for male recipients, small donor hearts, CVA as cause of death, donors with cancer history, smokers, diabetes and hypertension, ABO incompatibility, and ACCEPT, LV dysfunction in donor hearts, LVH, drugs users, and Hepatitis B&C [26].

Opinion and suggestions 

-HCV-positive and COVID positive hearts will substantially increase the number of available donor hearts.

-Should be more accepting of hearts from older donors aged 50 to 60 years old, after careful vetting.

-New allocation system is associated with higher transplantation, lower waitlist days and lower waitlist mortality. However, blood group O still needs more attention.

-A more general acceptance of ODDs and other drug overdose–related deaths for heart donation will further expand the donor pool.

## 3. Using Repairable Donor Hearts

**Fig. 2.**
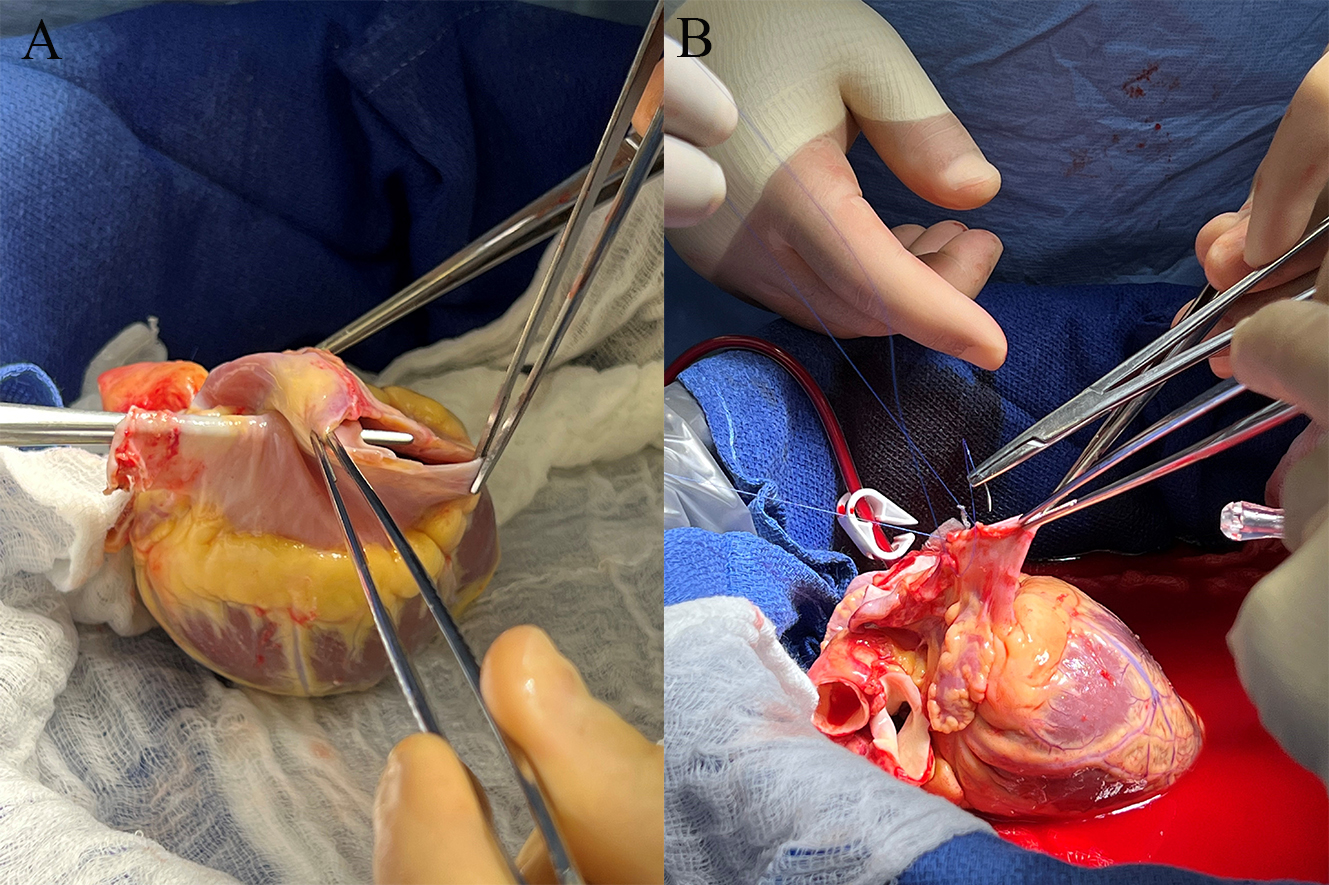
**Using repealable heart for transplantation**. (A) Back table inspection of a heart with congenital anomaly a single left Superior vena cava. (B) Back table repair and closure of the superior vena cava.

For a long time, donor hearts with associated valvular or discrete CAD have been considered unusable for heart transplant. However, with increasing demand for donor hearts and patients dying on the waitlist, repairing or replacing the diseased valves in the donor heart prior to transplant is a lucrative option. Further, this may be a viable option for patients with infective ventricular assist devices (VADs), those with heart failure who are not candidates for VAD, those from resource-limited countries, and those in rural or limited resource areas who urgently need a heart transplant. The first successful donor heart mitral valve repair followed by transplantation was published in 1996 [27]. Since then, numerous case reports and series have published their experience with mitral valve repair, aortic valve replacement, and coronary artery bypass grafting in the donor heart [28, 29, 30, 31, 32, 33, 34, 35, 36, 37, 38]. There are, however, genuine concerns for the durability and viability of replaced valves in the donor heart, as well as increased duration of ischemia when performing valve replacement prior to heart transplant [39]. The present experience with valvular heart surgery prior to transplant is limited to case reports and small case series with short-term follow-up. Until the availability of long-term results, use of these hearts for transplant should be decided on a case-to-case base. However, easily reparable congenital heart disease should not be a contraindication to accepting a heart if everything else is in normal formation (Fig. 2).

Opinion and suggestions 

-Bench aortic valve and mitral valve repair and replacement in donor hearts increases the donor pool. However, ischemic time should be carefully monitored.

-CABG surgery on donor hearts preferably done post cross clamp removal.

-Easily reparable congenital heart disease should not be a contraindication to accepting a heart if everything else is in normal formation.

## 4. Donation After Circulatory Death

A heart transplant from a DCD donor is not a new procedure. The first heart transplant done by Christian Bernard in 1967 was from a DCD donor. However, after the establishment of criteria for brain dead in the US and Europe in 1968, all heart donors were donation after brain death (DBD) for nearly 36 years [40]. However, this has been changing over the last 20 years. In 2018, about 20% of organ donations in the US were from DCD donors [41, 42]. Despite the large number of DCD heart donations, general acceptance is slow as there are concerns about graft functionality from DCD donors. In DCD donors, during the withdrawal of life-sustaining therapy, the heart undergoes a period of warm ischemia [43]; the concern is identifying the time point for myocardial dysfunction that can lead to graft dysfunction. Our current understanding is still limited about the duration after which irreversible myocardial cell damage occurs [44]. However, in a recent study, withdrawal of life-sustaining therapy appeared to maintain myocardial contractility and cellular viability for the first 10 minutes following cardiac arrest. Beyond this point, graft function was likely compromised [45].

DCD donors have increased the number of abdominal organs and lungs available for transplant, but the impact on heart transplants is not where it could be. Noterdaeme *et al*. [45] demonstrated that DCD hearts that met their criteria (DBD criteria + donation withdrawal ischemia time less than 30 minutes) could increase the number of heart transplants by 11% and reduce a single hospital’s waiting list death rate by 40% [46]. In another estimate by Messer *et al*. [42], DCD hearts have the potential to increase the heart transplant pool by 30% [47].

After the determination of circulatory death, there are few options to preserve the function of already ischemic hearts. One option is to start the heart in the donor thoracic cavity and convert the procurement to DBD, the other is to recover the heart and start it outside the body on an organ care system (OCS).

### 4.1 Normothermic Regional Perfusion

**Fig. 3.**
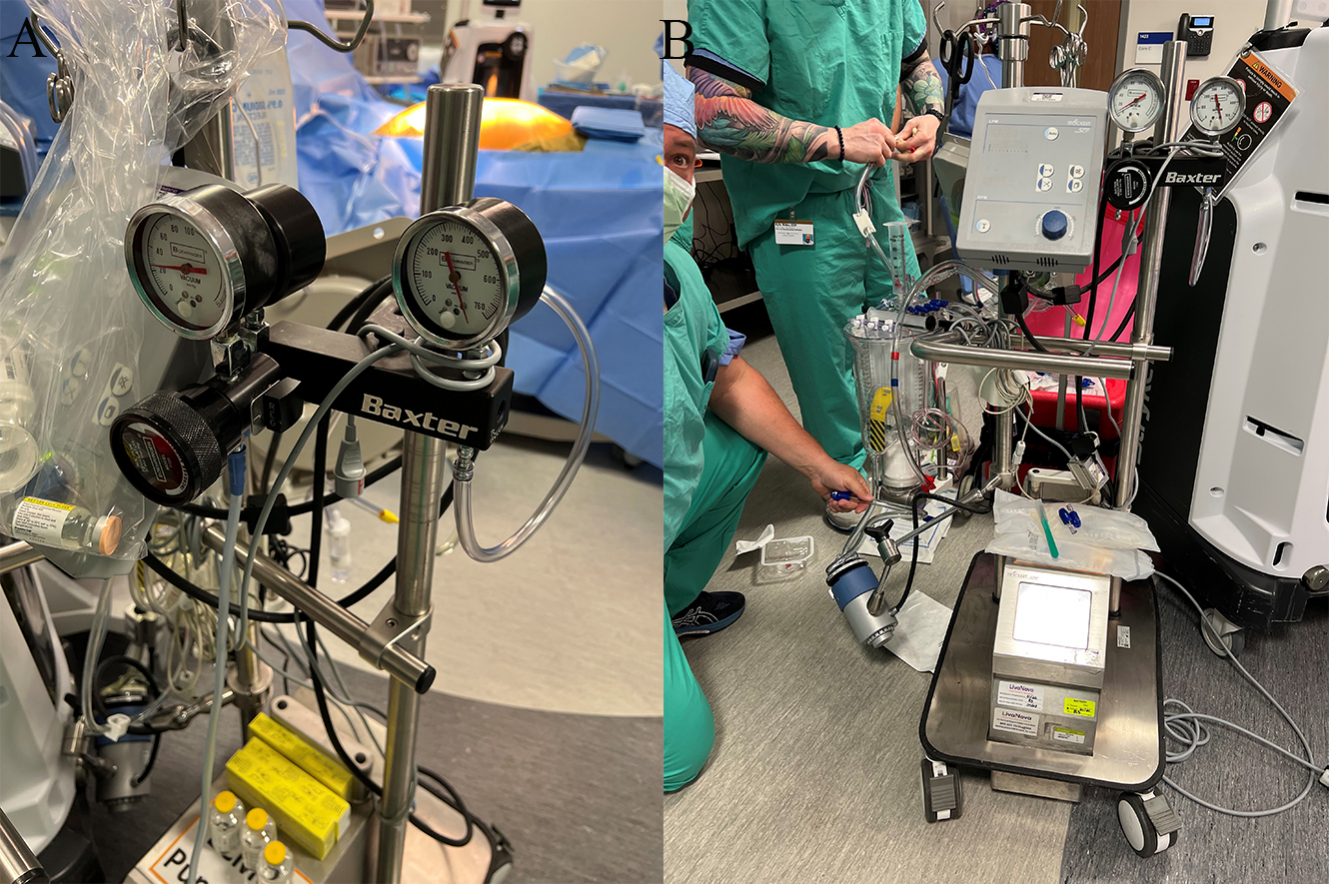
**Using NRP for organs recovery**. (A) Normothermic regional perfusion system. (B) Adding extra suction power.

Normothermic regional perfusion (NRP) involves the establishment of cardiopulmonary bypass via right atrial and aortic root cannulations following administration of 30,000 units of heparin and initiation of central venoarterial extracorporeal membrane oxygenation (Fig. 3). At the same time, head and neck vessels are cross clamped to prevent brain circulation. After the heart regains good contractility, the donor is weaned from extracorporeal life support to evaluate heart activity [48]. NRP will also establish blood flow to the abdominal organs, maintaining their vitality until the heart recovers. Besides its cardiac benefits, it reduced warm ischemia time for other organs and allows assessment the organs under a nonischemic condition (compared to cold storage). NRP can reduce cholangiopathy in the liver [49] and can affect earlier recovery in kidney transplantation compared to in situ cold perfusion [50].

Another method of harvesting DCD hearts is direct procurement and perfusion (DPP). In this technique, the chest is entered in the shortest possible duration, the aorta is cross clamped, and cardioplegia is delivered in the aortic root. Compared to NRP, this technique does not provide an in situ functional assessment of the donor organ and relies on an expensive *ex-vivo* perfusion platform, under physiological conditions. The vitality of the other organs is also at stake. However, Smith and colleagues [50] have demonstrated promising outcomes, including weaning all donor hearts off of cardiopulmonary bypass without inotropes, a 100% recipient survival rate with a median follow-up of approximately one year, post-discharge left ventricular ejection fraction of 64%, and no patients requiring mechanical circulation support [51].

We can use NRP in 2 techniques for DCD donor heart transplant: NRP followed by machine perfusion and NRP followed by cold storage [52, 53, 54]. Previously, DCD heart transplantation was limited by the requirement for the donor and the recipient to be in the same location, as in the first heart transplant. However, with the use of NRP, we can transport the heart longer distance [55].

DPP induces cardiac arrest through cannulation and delivery of cold cardioplegia, after cross-clamping, cold ischemic time begins. If *ex-vivo* organ perfusion is to be performed, autologous blood needs to be removed from the donor before cardioplegia is delivered [56]. In contrast to DBD hearts or DCD hearts procured by NRP, no evaluation of cardiac function can be done prior to transplantation and release of aortic cross clamp. Messer and colleagues, comparing the outcomes between DCD heart transplants performed with DPP and NRP, found no significant difference between the two [57]. Spoga *et al*. [58] study cold storage versus normothermic perfusion organ preservation showed that using of *ex-vivo* graft perfusion in patients on mechanical circulatory support improve the outcome post heart transplant.

However, due to a paucity of studies, this should be investigated further.

DCD heart transplantation with NRP needs to overcome several important obstacles before it can become a mainstream procedure. Resolving these concerns could substantially increase the heart transplant donor pool. First, NRP with cardiopulmonary bypass is logistically challenging, requiring considerable coordination between the donor hospital, procurement teams, perfusionists, and organ procurement organization for successful implementation. Secondly, as donor and recipient location is an important concern for DCD hearts [59], *ex-vivo* machine perfusion or cold storage is necessary to relocate the allografts.

Ethical issues are a concern for every procedure, but especially for DCD and DBD. Some countries, such as Australia, prohibit the practice of DCD. As the debate around the ethical aspects of NRP heart procurement continues, it is of paramount importance that clear communication takes place with donor families to avoid misunderstandings and preserve trust.

While DCD heart transplantation can increase the pool of heart donors, there are not many studies that compared NRP to DPP and *ex-vivo* reperfusion. Comparison, mortality rates, and adverse effects must be investigated in further studies to identify the benefits and disadvantages of these techniques.

### 4.2 OCS for Heart Transplant

**Fig. 4.**
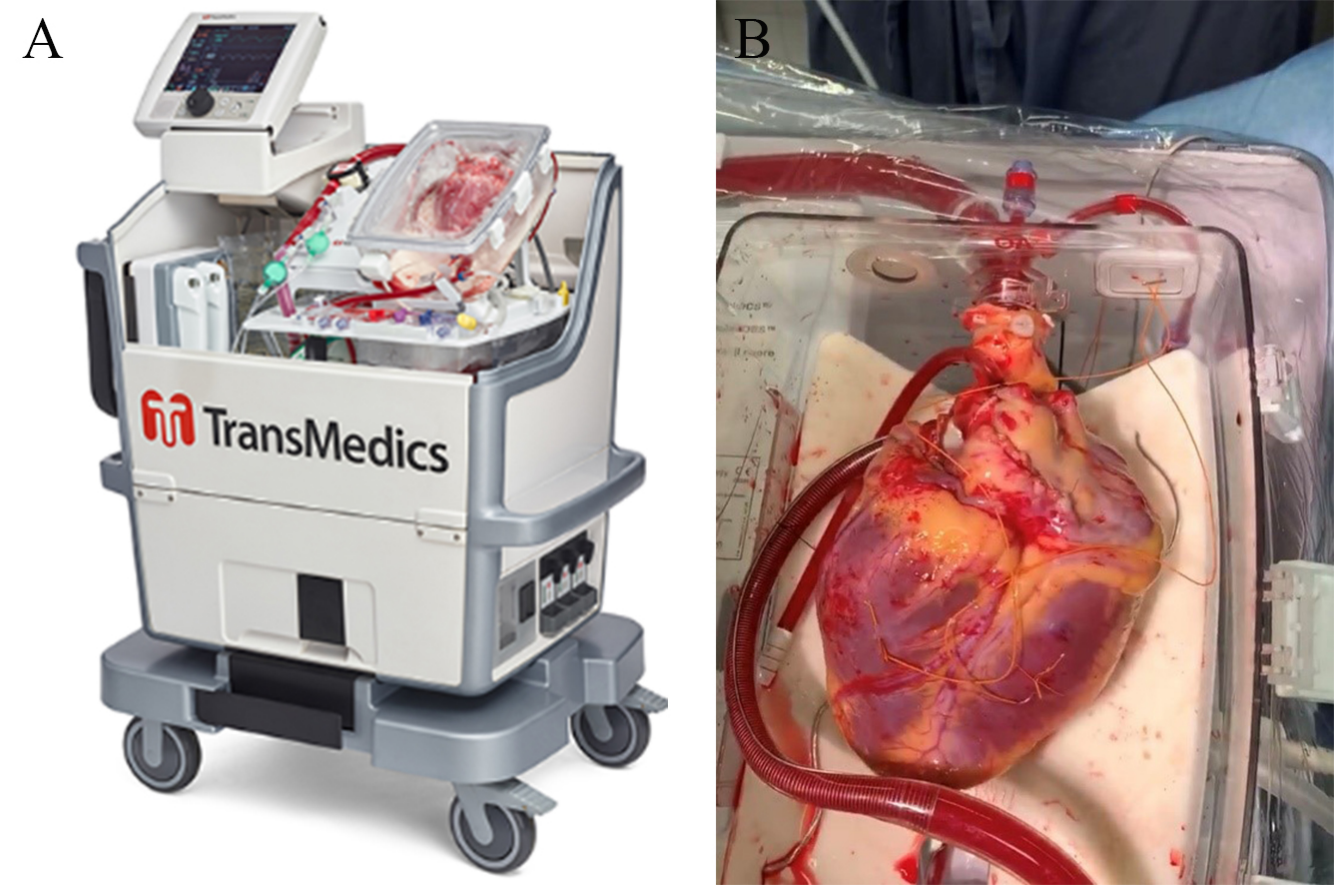
**Organ care system for donation after circulatory death**. (A) The portable OCS device with all parts. (B) Aortic and pulmonary artier connections to the device .

OCS for the heart is a portable extracorporeal heart perfusion and monitoring system indicated for the resuscitation, preservation, and assessment of donor hearts in a near-physiologic, normothermic, and beating state intended for a potential transplant recipient (Fig. 4). It allows continuous monitoring of aortic pressure, lactate level, and coronary blood flow of the graft [60]. Continuous measurement of lactate levels from the arterial and venous side of the graft in the OCS allows assessment of the adequacy of coronary blood flow [61].

In comparison to static cold storage (SCS), OCS technology can preserve the heart for a longer duration of time and significantly reduces the total cold ischemia time. Cold ischemia time in OCS is limited to the initial and final phases of donor heart procurement (i.e., prior to connecting the heart to OCS and after disconnecting the heart from OCS and until the release of the aortic cross clamp) [62]. In the first 30 to 40 years of heart transplantation, all emphasis was on the management of the recipient, and the donor heart was considered an organ that could be safely stored and transported in cold solution for any duration. Therefore, only good hearts were procured for transplantation. The introduction of OCS has been a game changer in this respect as longer preservation time allows us to examine and manage the donor graft in real time before transplant, which may increase the donor pool with marginal hearts [63].

Total ischemia time of the heart and older age of the donor are major risk factors for mortality after heart transplant. One study showed that mortality at 1 year posttransplant doubles when the cold ischemic time is increased from 3 hours to 6 hours and halved when it is less than 1 hour [64]. Increased cold ischemia time is associated with increased risk of ischemia reperfusion injury and primary graft dysfunction, while increased donor age is associated with greater risk of CAD in the heart. OCS markedly decreases the cold ischemia time and allows angiography, which is the criterion standard method to definitively diagnosis CAD. Moreover, rising aortic pressure during perfusion of the donor heart in OCS is an indirect predictive indicator of CAD. This is important because, when angiography is not available, monitoring aortic pressure and lactate can aid in determining the severity of CAD and subsequently the viability of the graft [65].

PROCEED II trial a prospective multicenter study demonstrated for the first time the outcome of heart grafts preserved with OCS heart system was comparable to those preserved using SCS [62]. Recipient 30-day outcomes and graft survival were not affected with the use of the OCS for heart preservation when compared to SCS. Moreover, there was no significant difference in the incidence of severe rejection and length of stay in the intensive care unit between the patients who had a graft preserved using OCS compared to SCS [57]. A retrospective single-center review by Kaliyev *et al*. [66] also showed better outcomes for patients who underwent transplant using an OCS heart compared to SCS.

Another area where OCS has an important application is heart transplant in patients with congenital heart disease. Post–heart transplant mortality for patients with congenital heart defect is very high, especially for those who have undergone previous surgical intervention. It remains a challenge for surgeons to explant the heart and delineate the complex anatomy in the presence of severe adhesions from the previous surgery. A study by Fleck *et al*. [67] suggested that pediatric transplant patients may have better outcomes when the heart is perfused with OCS than when SCS is used. Patients who have VADs may benefit from OCS because their anatomic complexity is greater and preparation for transplantation can be harder (increasing time of cold ischemia). A small retrospective study suggested that use of OCS before heart transplant in patients with VAD had better 30-day survivability when compared to SCS [68]. However, due to the limited sample size, more research using OCS is needed to explore its application and outcome.

Despite considerable progress in the field of heart procurement, DCD still remains a challenge, and many institutions are working to create a novel method of DCD heart preservation that reduces further ischemic insult, provides a platform for organ resuscitation, and allows for graft viability testing prior to transplantation following an unavoidable warm ischemic injury. Iyer *et al*. [69] demonstrated that DCD hearts are a viable option for transplantation despite high warm ischemic insult if managed by OCS system.

Using OCS will help reach patients located far from the donor heart site to receive heart transplantation [70]. OCS allows use of DCD hearts, which can increase the number of hearts available and decrease patients’ time on the waiting list for heart transplant.

Opinion and suggestions

-After the determination of circulatory death, there are few options to preserve the function of already ischemic hearts. One option is to start the heart in the donor thoracic cavity and convert the procurement to DBD by using NRP system, the other is to recover the heart and start it outside the body on *ex-vivo* such as organ care system (OCS).

-The first option is more cost effective. However, other organs depend on successfully establishing the system and short time.

-The second option is more expansive. However, failure of the attempt reflect only on the heart.

-Both options needs mobilization of a great deal of resources.

## 5. Xenotransplantation 

Xenotransplantation is transfer of organs across species. Although, folklore in various civilizations is filled with stories of multiple and complex xenotransplant, reality tells a different story [71].

People began experimenting with xeno-blood transfusion, teeth, and skin grafts in humans as early as the 17th century with minimal success [72, 73, 74]. Multiple attempts at nonhuman primate and mammalian organ transplantation in humans ended in graft failure due to acute graft rejection and vascular thrombosis [75, 76, 77, 78]. Despite multiple immunosuppressive drugs and full body radiation, the longest xenotransplant survival achieved was 9 months [79].

Table 1 (Ref. [80, 81, 82, 83, 84, 85, 86, 87, 88, 89]) displays landmarks in xenotransplantation since the first xeno-heart transplant by James Hardy in 1964. He transplanted a chimpanzee’s heart into a 68-year-old man with heart failure, shock, and left leg gangrene.

**Table 1.** **The main stepping stones of xenotransplantation through out last 5 decades**.

Year	Surgeon (location)	Donor organ and source	Survival	References
1964	James Hardy (US)	Chimpanzee heart	90 minutes	[80]
1968	Donald Ross (UK)	Pig heart	4 minutes	[81]
1968	Denton Cooley (US)	Sheep heart	10 minutes	[82]
1969	Pierre Marion (France)	Chimpanzee heart	“quickly”	[83]
1977	Christian Barnard (South Africa)	Chimpanzee heart	4 days	[84]
1984	Leonard Bailey (US)	Baboon heart	20 days	[85]
1992	Zbigniew Religa (Poland)	Pig heart	23 hours	[86]
1996	Dhaniram Baruah (India)	Pig heart	7 days	[87]
2022	Bartley Griffith (US)	Pig heart	60 days	[88, 89]

However, first short-term success in xenotransplantation was achieved recently when in January 2022, Dr. Barley P. Griffith and his team at the University of Maryland Medical Center transplanted a genetically modified pig heart into a patient suffering from terminal heart failure [90]. Altogether, 10 genes in the pig heart were altered to prevent acute graft rejection [91, 92, 93, 94, 95, 96]. However, death of the recipient at 2 months after transplant has again raised questions about the future of xenotransplantation. On the other hand, Dr. Griffith’s success, though short, makes xenotransplantation seem closer to reality. This case gives hope that with further understanding of xenotransplant graft rejection and improvement in bioengineering, xenotransplantation may one day be possible.

Social, zoonotic, ethical, moral, and religious issues will remain major obstacles to xenotransplantation [97, 98, 99]. Also, issues of transmission of zoonotic diseases from animal to human have yet to be analyzed. Pigs are reservoirs for many viral pathogens, such as hepatitis E virus, cytomegaly virus, and porcine lymphotropic herpesviruses, and many retroviruses. Whether xenotransplantation increases the risk of these zoonotic diseases is yet to be seen [100].

Opinion and suggestions

Social, zoonotic, ethical, moral, and religious issues will remain major obstacles to xenotransplantation. Also, issues of transmission of zoonotic diseases from animal to human have yet to be analyzed. More research effort needed. Human recipients’ engagement is encouraged and safe setting and acceptable ethics.

## 6. Bioengineered Hearts from Recipient Tissue

Development in the field of genetics and human genome project has paved the way for bioengineering of human tissues. Although, human tissue as been successfully bioengineered the laboratory, the field of bioengineering is still in its infancy, and successful growth of an artificial heart is still long way off. This section aims to discuss the advances that have already been made and the future challenges of bioengineering a human heart suitable for transplantation.

### 6.1 Anatomical Scaffolds 

The heart is a framework of anatomical scaffolds that support the specific functions of cells organized into structures such as vessels, muscles, and nerves. These scaffolds consist of collagen, laminins, polysaccharides, and peptidoglycans embedded in a matrix of complex sugars and chemokines, which allows optimal coordination of the mechanical and electrical functions of the heart [101, 102]. The first challenge is to construct a scaffold around which the specialized cells can be grown and maintain their viability through blood perfusion [98].

### 6.2 Decellularization 

Researchers have been able to develop the technique of decellularizing the tissue while retaining its composition, architecture, and mechanical properties [103, 104]. Decellularized tissue provides a dynamic environment for the orientation and coupling of cells and facilitates the exchange of nutrients and oxygen throughout the depth of the tissue. This process also removes a majority of both allogeneic and xenogeneic antigens, which may prevent the need for immunosuppressants [105]. Animal and human heart decellularized extracellular matrix (ECM) scaffolds also remove much of the antigen load [106]. However, the porcine heart ECM contains α-1,3-galactose epitope, which can stimulate an immune response [107, 108]. A cadaveric heart that is unfit for transplantation can be used to harvest an ECM scaffold [109]. The only drawback is that it may not always be possible to achieve the desired level of tissue engineering fidelity with these matrices because they may be damaged or diseased. To circumvent these issues, further research is needed [110, 111, 112]. Researchers have also studied various synthetic scaffolds as potential surrogates for the ECM, but none can replicate its intricacy or structure compared to native ECM.

### 6.3 Recellularization

To achieve a functional organ, recellularization of the scaffold with cells from fetuses and adults, such as embryonic, mesenchymal, and induced pluripotent stem cells, has been tried with limited success [113, 114]. The major issue with recellularization of the scaffold is absence of uniformity, which leads to thrombogenesis and arrhythmogenesis [115] in the heart tissue. The potential problem with intramyocardial injections was that even though the injection site showed dense cellularity, the cells were poorly distributed throughout the scaffold [116].

Cell seeding techniques for the heart usually involve seeding by perfusion through the vascular tree. Improved cell concentration and diffusion over the scaffold can be achieved by optimizing the mechanical environment, scaffold coating, and cell perfusion systems by using multiple perfusion routes, which for the heart involves both direct intramyocardial injections and perfusion of the vascular tree [117]. After enough cells have been seeded onto an organ scaffold, cell culture is required. A bioreactor is necessary for perfusion and provides a nutrient-rich environment that encourages organ-specific cell growth [98]. Bioreactors should allow nutrient-rich oxygen to be pumped with adjustable rates of flow and pressure, as well as monitor and control the pH and temperature of the media [117].

By implementing these methods, heart constructs engineered with progenitor cells not only generate mechanical force, but also exhibit responsiveness to drugs and electrophysiological characteristics. However, electrocardiogram analysis of the bioengineered hearts has also shown irregular wave morphology due to loss of coupling between cardiomyocytes [118].

Opinion and suggestions

To achieve a functional organ, recellularization of the scaffold with cells from fetuses and adults, such as embryonic, mesenchymal, and induced pluripotent stem cells, has been tried with limited success.

Further research must be conducted until a mechanically, electrically, and physiologically well-coordinated organ can be constructed and ultimately transplanted into human patients.

## 7. Conclusions

In the US, more than 40,000 solid organ transplants are done annually, including more than 3000 hearts. To meet this demand, there is an urgent need to increase the potential donor pool. Presently, less than 50% of potential organ donors become actual donors. In the last decade, there have been several attempts to expand donor criteria to increase the donor pool. But despite expanding donor criteria and new technologies for donation after circulatory death (DCD) heart and donor heart repair, the gap between supply and demand is widening and unfortunately, thousands die every year waiting due to the critical shortage of organs. Furthermore, even with continuous advancements in regenerative medicine and xenotransplantation, among other, to combat the need for heart transplant, the shortage of heart donors will continue to grow. Perhaps, more effective strategies are needed.
